# Supplementary material for: Synthesis and Properties of New Multilayer Chitosan@layered Double Hydroxide/Drug Loaded Phospholipid Bilayer Nanocomposite Bio-Hybrids
Source: Materials (Basel). 2020 Aug 12;13(16):3565. doi: 10.3390/ma13163565 (PMC7476003; doi:10.3390/ma13163565)
Supplement: Supplementary file 1 [file materials-13-03565-s001.pdf]

materials

Synthesis and Properties of New Multilayer Chitosan@layered Double Hydroxide/Drug Loaded Phospholipid Bilayer Nanocomposite Bio-hybrids

Dan A. Lerner [1,*], Sylvie Bégu [1], Anne Aubert-Pouëssel [1], Ramona Polexe [1], Jean-Marie Devoisselle [1], Thierry Azaïs [2] and Didier Tichit [1,*]

[1] ICGM, University of Montpellier, CNRS UMR 5253, ENSCM, 34296 Montpellier, France; sylvie.begu@enscm.fr (S.B.); anne.aubert@univ-montp1.fr (A.A.P.); ramona_polexe@yahoo.com (R.P.); jean-marie.devoisselle@enscm.fr (J.M.D.)

[2] Laboratoire de Chimie de la Matière Condensèe de Paris (LCMCP), Collège de France, Sorbonne Université, CNRS, F-75005 Paris, France; thierry.azais@sorbonne-universite.fr

[*] Correspondence: dan.lerner@enscm.fr (D.A.L.); didier.tichit@enscm.fr (D.T.)

Received: 20 July 2020; Accepted: 10 August 2020; Published: date

Supplementary Information

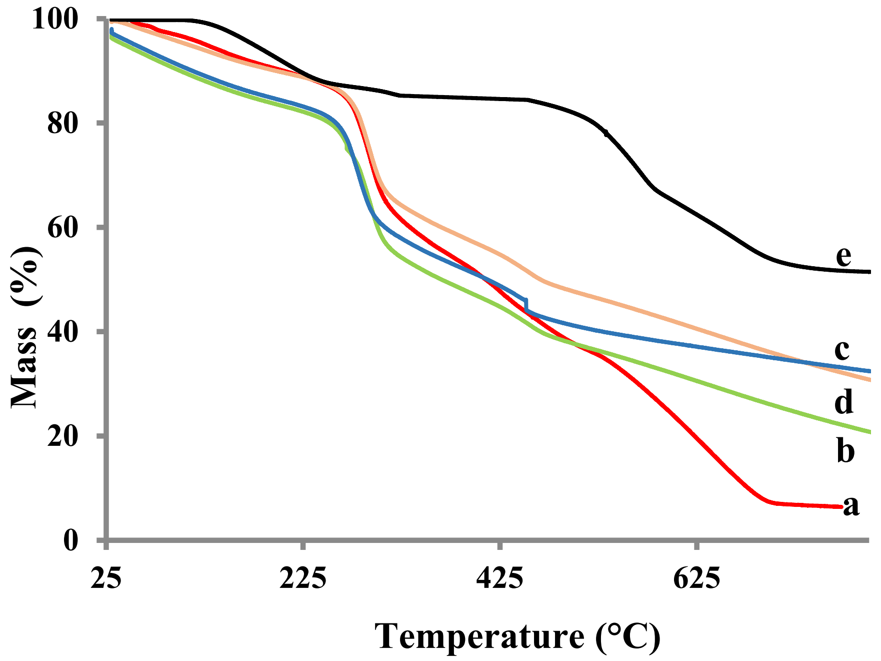

Figure S1. TG profile of pure C (**a**), C-LDH(2) beads (**b**), C-LDH(1) beads (**c**), C-LDH(0.5) beads (**d**) and Mg/Al-NO_3 LDH (**e**).

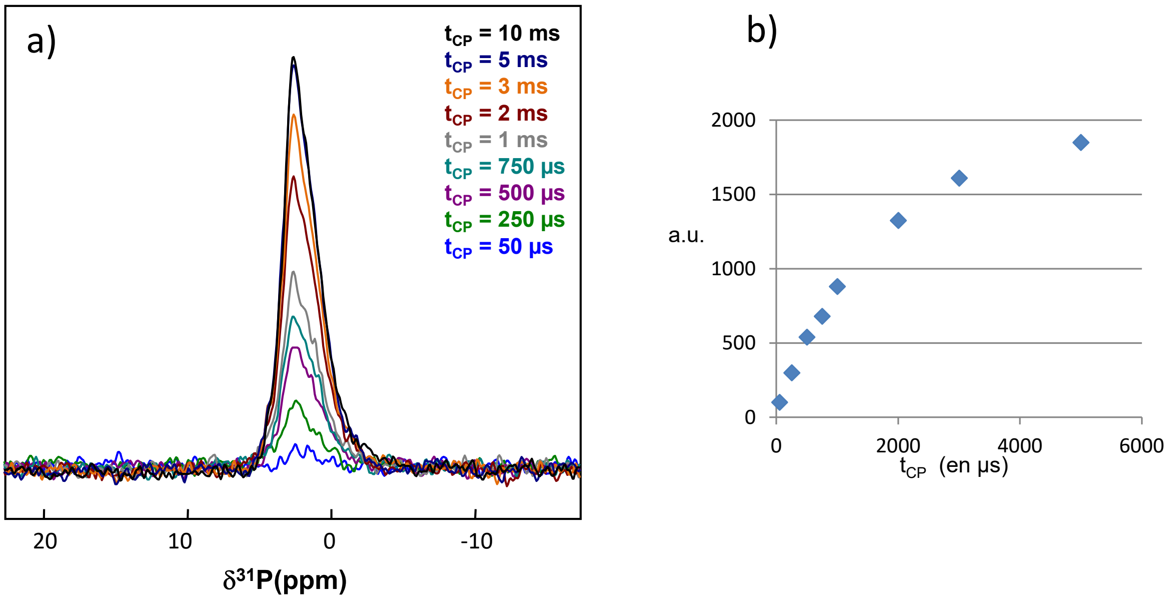

Figure S2. (**a**) Variable contact time ^{31}P CP MAS experiment for LDH-BL and (**b**) the corresponding evolution of the ^{31}P signal intensity as a function of the contact time

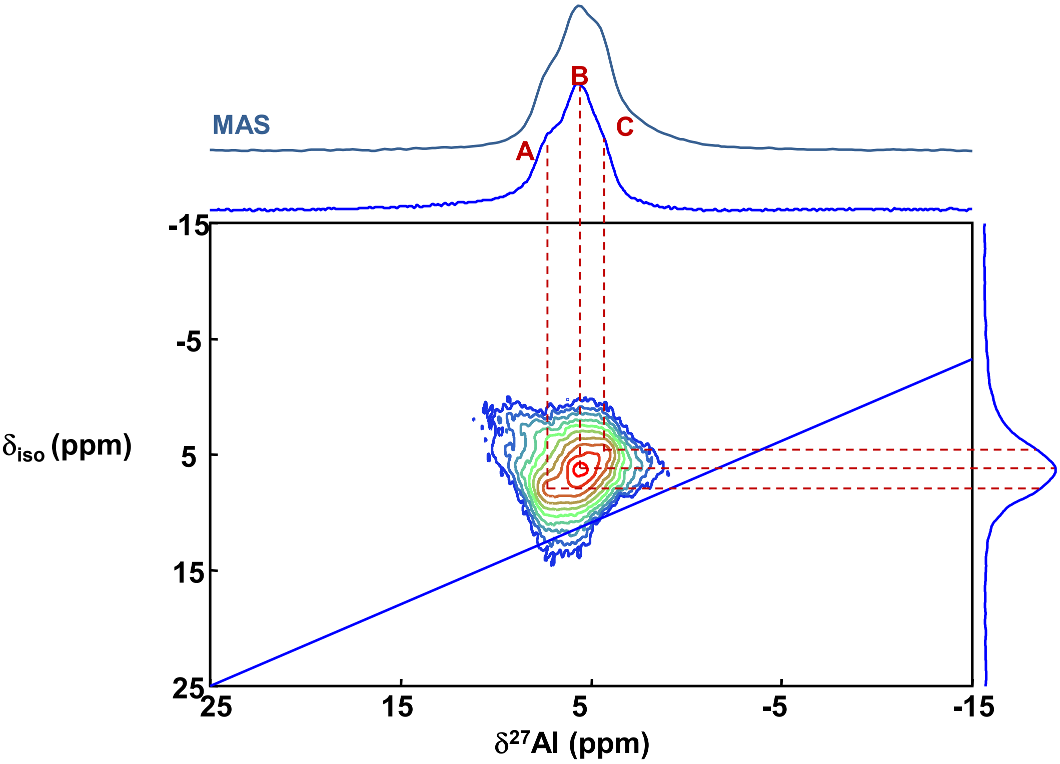

Figure S3. ^{27}Al MQ MAS NMR spectrum of LDH-BL

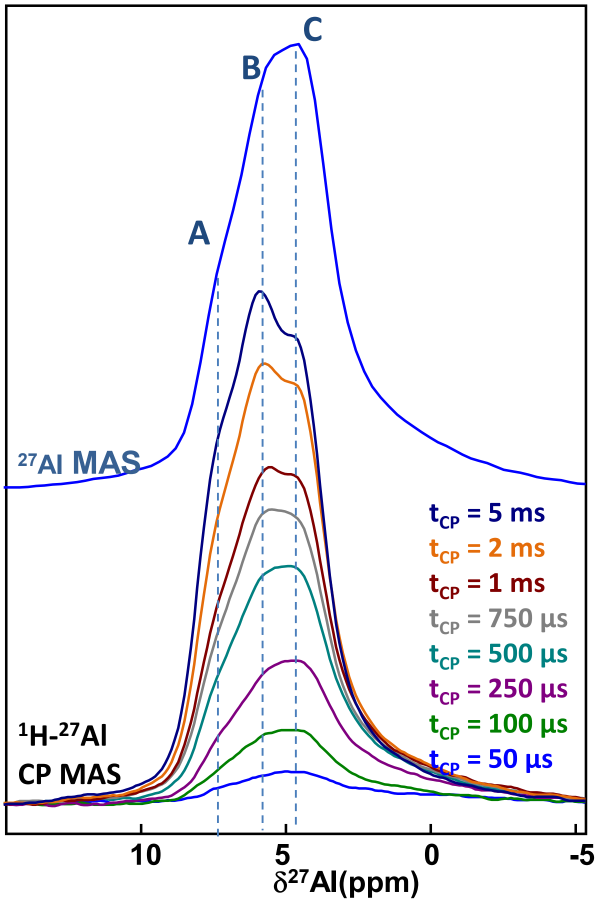

Figure S4. One dimensional ^{1}H-^{27}Al CP MAS spectra of LDH-BL recorded at various contact times and comparison with the ^{27}Al MAS spectrum.

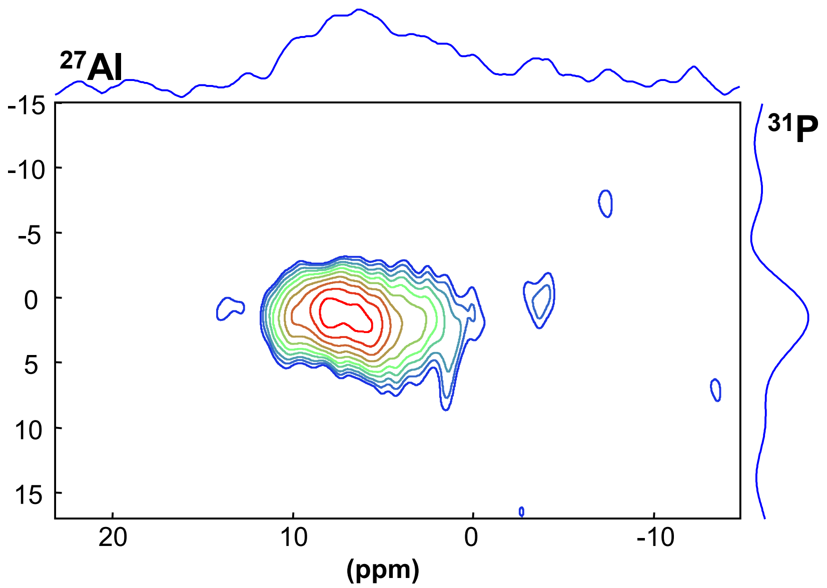

Figure S5. Two dimensional ^{27}Al-^{31}P CP MAS spectrum (t_{CP} = 2.5 ms) of LDH-BL
